# Mapping biological entities using the longest approximately common prefix method

**DOI:** 10.1186/1471-2105-15-187

**Published:** 2014-06-14

**Authors:** Alex Rudniy, Min Song, James Geller

**Affiliations:** 1 Computer Science Department, New Jersey Institute of Technology, Newark, NJ 07102, USA; 2 Department of Library and Information Science, Yonsei University, 50 Yonsei-ro, Seoul 120-749, Korea

## Abstract

**Background:**

The significant growth in the volume of electronic biomedical data in recent decades has pointed to the need for approximate string matching algorithms that can expedite tasks such as named entity recognition, duplicate detection, terminology integration, and spelling correction. The task of source integration in the Unified Medical Language System (UMLS) requires considerable expert effort despite the presence of various computational tools. This problem warrants the search for a new method for approximate string matching and its UMLS-based evaluation.

**Results:**

This paper introduces the Longest Approximately Common Prefix (LACP) method as an algorithm for approximate string matching that runs in linear time. We compare the LACP method for performance, precision and speed to nine other well-known string matching algorithms. As test data, we use two multiple-source samples from the Unified Medical Language System (UMLS) and two SNOMED Clinical Terms-based samples. In addition, we present a spell checker based on the LACP method.

**Conclusions:**

The Longest Approximately Common Prefix method completes its string similarity evaluations in less time than all nine string similarity methods used for comparison. The Longest Approximately Common Prefix outperforms these nine approximate string matching methods in its Maximum *F*_1_ measure when evaluated on three out of the four datasets, and in its average precision on two of the four datasets.

## Background

The term-matching problem has been widely addressed in multiple contexts, which resulted in a number of string similarity metrics designed, applied and evaluated in various research studies [1]. In the biomedical domain, various ASM methods are used by scientists to solve current research tasks such as retrieving sequences from existing databases that are homologous to newly discovered ones, and establishing multiple sequence alignment to discover similarity patterns to predict the function, structure, and evolutionary history of biological sequences [2].

The recent expansion of healthcare information systems that draw from multiple medical databases has resulted in redundant information, among other problems. This phenomenon, also known as the duplicate detection problem, has caused problems with record linkage across medical databases. Previous research has addressed problems such as patient record aggregation from multiple databases based on a minimum profile (i.e., name, gender and date of birth) [3] and term matching for source integration, spelling correction and biomedical data mining applications. In this paper, these tasks are considered in the context of terminologies such as Systemized Nomenclature of Medicine Clinical Terms (SNOMED CT) and the Unified Medical Language System (UMLS) [4]. Approximate String Matching (ASM) methods are used for augmenting, updating, and auditing UMLS vocabularies. ASM methods are also important for facilitating biomedical information extraction, relationship search, and concept discovery [5].

The UMLS is an extensive terminological knowledge base comprised of three major components: the Metathesaurus, the Semantic Network, and the SPECIALIST Lexicon and Lexical Tools. The current 2013AB release of the Metathesaurus contains more than 2.9 million concepts and 11.4 million unique terms retrieved from over 160 source vocabularies [6]. UMLS source integration is a complicated multistep process and, despite the availability of numerous algorithmic tools, managing these vocabularies requires considerable human involvement. As additional sources are integrated into the UMLS, they will require reintegration with existing vocabularies [4].

These disadvantages motivate the search for a new method for approximate string matching and UMLS-based evaluation. In this paper, we introduce the Longest Approximately Common Prefix (LACP) method for ASM and present the results of its use to improve the operation of a number of applications in biomedical informatics and related domains.

It bears noting that, in contrast to the well-known SPECIALIST lexicon tools Norm, Word Index or LVG [7], LACP does not perform text manipulations. Instead, it assesses the similarity or dissimilarity of two strings.

Other three highly praised instruments, MetaMap [8], NCBO Annotator [9] and ConceptMapper [10] are publicly available concept recognition systems designed for text annotation from various ontologies [11]. The general rationale of these tools is to split the input text into smaller constructions, such as phrases or tokens, which are subsequently looked up in a dictionary. For instance, MetaMap splits the input text into phrases and produces their variants. Then it generates a candidate set, which is mapped to an ontology. The LACP method, introduced in this paper, may be used as an inner component of such a system for calculating the similarity of a candidate phrase or token when matching to various ontology terms. The authors consider implementation of a text annotation system incorporating the LACP method as a direction for future research.

The rest of the section is dedicated to the analysis of the relevant research approaches and the related work studying the application of well-known similarity measures in the biomedical domain.

Tan et al. [12] applied the classic Levenshtein score incorporated with a particular threshold to medical ontology alignment. Tolentino et al. [13] utilized the Levenshtein technique in combination with other string similarity algorithms to construct a UMLS-based spell checker. Sahay et al. [14] employed more advanced combinations of the Jaro and Jaro-Winkler similarity metrics combined with Term Frequency/Inverse Document Frequency (TFIDF) to compute similarity values between ontological concepts and phrases. Cohen et al. [15] described, implemented and evaluated the above-mentioned hybrid distances in the SecondString Java toolkit.

Plaza et al. [16] applied heuristic rules with a clustering algorithm to the problem of biomedical text summarization. Their work mapped terms found in a given document to UMLS concepts. Using the relationships between the identified UMLS concepts, the authors then represented the document in a graph. They graphed the concepts and assigned sentences to clusters based on semantic similarity. Finally, the most important sentences were selected to be included in a document summary.

Zhen et al. [17] introduced a TFIDF string distance method within their clustering algorithm and applied it to biomedical ontologies. The evaluation of their method demonstrated superior values of the *F*-measure on two datasets derived from the MeSH and GO ontologies.

In a previous paper, we developed a novel Markov Random Field-based Edit Distance (MRFED) and applied it to the ASM problem in GO ontologies [18]. Similarly, Wellner et al. [19] used Conditional Random Fields in a distance metric method on a UMLS Metathesaurus dataset. Bodenreider et al. [20] applied the Cosine, Jaccard and Dice string similarity coefficients to aligning the UMLS Semantic Network with the Metathesaurus.

Yamaguchi et al. [21] tested four similarity metrics for clustering terms, which appeared in the UMLS Metathesaurus. The authors compared the performances of Monge-Elkan, SoftTFIDF, Jaro-Winkler and the bigram Dice coefficient methods evaluating these techniques on chemical and non-chemical terms grouped into two datasets. They demonstrated that normalized string distances performed better than the standard measures for the evaluation of precision, recall, and *F*-measure, and that similarity metrics required different parameters such as threshold values for chemical and non-chemical terms, among other findings.

Sauleau et al. [22] propose a novel method for linking medical records by examining the connections between stand-alone and clustered databases. The authors developed a three-step approach: 1) preprocessing the data and applying blockers, 2) matching pairs of records using the Porter-Jaro-Winkler score calculation, and 3) clustering the data. The authors suggest that their method is useful for inserting new entities into large databases.

Zunner et al. [23] studied the semi-automated mapping of non-English terms to Logical Observation Identifiers Names and Codes (LOINC) [24] using the Regenstrief LOINC Mapping Assistant (RELMA) [25]. Their approach resulted in a mapping rate of 500 terms per day, which they considered satisfactory.

In research by Parcero et al. [26], mapping a local terminology to the LOINC dataset led to the development of an automated tool that uses an approximate string matching function. McDonald et al. benchmarked Jaccard, Levenshtein, Monge-Elkan, and Soft TFIDF metrics for LOINC integration, and the Jaccard method was selected as the best choice for such a task [24].

The present research employs the Shortest Path Edit Distance (SPED) algorithm we developed previously [27] to compute a string distance based on substring matching and graph-based transformations. To adjust the dissimilarity values in the final results, we applied a re-scorer set according to the length of equal string prefixes. This final step produced a major improvement in results and inspired this paper on the Longest Approximately Common Prefix (LACP) method, a novel string similarity metric based on the approximate prefix match of two strings. This paper demonstrates how this fast string distance method provides performance that is superior to other methods on datasets from SNOMED CT and from multiple UMLS sources (Table 1) in terms of average precision and Maximum *F*_1_.

**Table 1:** Four medical informatics datasets used in experiments

**#**	**Dataset**	**# of concepts**	**# of terms**	**Size in kilobytes**
*D*_1_	The UMLS most frequent concepts from multiple sources	100	4,979	369
*D*_2_	The SNOMED CT most frequent concepts	155	5,000	281
*D*_3_	The UMLS concepts with longest terms (“longest concepts”)	3,337	5,000	1,693
*D*_4_	The SNOMED CT longest concepts	1,805	5,000	903

## Methods

The Longest Approximately Common Prefix (LACP) method is based on an approximate histogram match of string prefixes. It identifies matches by determining the similarity value of a pair of strings. The method compares the histogram differences between the prefixes of two strings to parameter α*.* It begins its search in the first characters of the strings. The prefix length is returned when the histogram difference is equal to α or the last character of the shorter string is reached. The prefix length is then divided by the average length of the pair of strings. The division takes into consideration string lengths, since strings that have significantly varying lengths are more dissimilar than strings that do not. The division also assures that the value of the LACP function stays in the [0, 1] interval. The formula for the LACP function (1) is as follows:

(1) $$\text{LACP}\left(S,T\right)=1-\frac{\text{prefLength}\left(S,T\right)}{\left(\left|S\right|+\left|T\right|\right)/2}$$

where *prefLength* is the length of the longest approximately common prefix. According to formula (1), for two identical strings, LACP is 0, whereas LACP is 1 for two strings not sharing any common prefix under a certain selection of the parameter α. The formula for *prefLength* is given in (2) below:

(2) $$\begin{array}{c} \text{prefLength}=\left\{i\left|\left(\text{prefHistDiff}\left(S_{1..i},T_{1..i}\right)\right)\right)\right\}\\ \;\left(\left(=\alpha \right)\cap \left(\text{prefHistDiff}\left(S_{1..i-1},T_{1..i-1}\right)<\alpha \right)\right\} \end{array}$$

where *prefHistDiff* is a histogram difference function of string prefixes, α is a parameter, and *S*_1..*i*_ and *T*_1..*i*_ are prefixes of strings *S* and *T* of length *i*. For example, for the strings *S* = Anorexia and *T* = Angina, with an α = 2, the *prefLength* would be 3, because two initial characters match and α allows only one mismatch. Alternatively, with α = 3 the *prefLength* would be 4 because two mismatches are allowed.

The histogram difference function for string prefixes is defined in formula (3):

(3) $$\text{prefHistDiff}\left(S_{1..i},T_{1..i}\right)=i-\left|\text{hist}\left(S_{1..i}\right)\cap \text{hist}\left(T_{1..i}\right)\right|$$

where *hist* is a histogram, and *i* satisfies the inequality (4):

(4) $$1\leq i\leq \text{min}\left(\left|S\right|,\left|T\right|\right)$$

A histogram is an array, that counts the number of occurrences of each distinct symbol in a string. In formulae (2) and (3), *i* denotes a prefix length. By subtracting from *i* the number of characters that are common to the histograms of both prefixes, the number of non-common characters remains in the difference. This number of non-common prefixes is matched against the parameter α, as is shown in formula (2). During the evaluation phase, we used α = 3, which allowed two mismatches in histogram difference*.*

**Figure 1:**
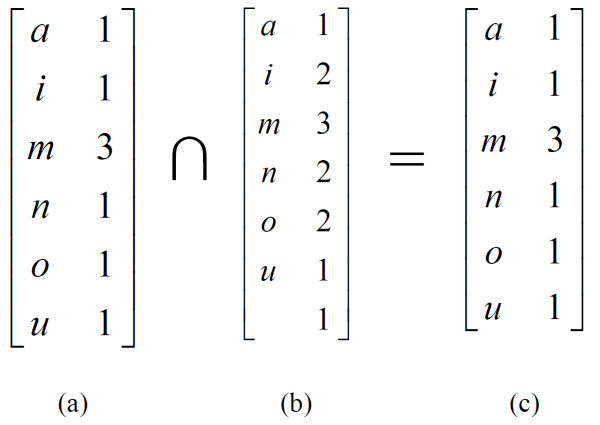
**Example of histogram intersection.** The expression *hist*(*S*_1..*i*_) ∩ *hist*(*T*_1..*i*_) denotes the histogram intersection of two string prefixes. Depicts the histogram intersection of two UMLS terms, *ammonium* and *ammonium ion*. The histogram of *ammonium* is in **a**, the histogram of *ammonium ion* is in **b**. The intersection **(c)** is computed as the minimum for each pair of argument values of the same character, with missing values in one argument omitted from the result. For example, *ammonium* contains one “o” while there are two letters “o” in *ammonium ion.* As min (1, 2) = 1, the resulting histogram in **c** contains the entry “1” for the letter “o.” As there is no blank in *ammonium,* there is also no entry for the blank character in the resulting histogram. In order to compute the size (the “absolute value” ||) of the histogram intersection in **c**, the sum of all the numbers in the result matrix is calculated. For **c**, the size of the histogram intersection is (1 + 1 + 3 + 1 + 1 + 1) = 8.

The expression *hist*(*S*_1..*i*_) ∩ *hist*(*T*_1..*i*_) denotes the histogram intersection of two string prefixes. Figure 1 depicts the histogram intersection of two UMLS terms, *ammonium* and *ammonium ion*. The histogram of *ammonium* is in Figure 1a, the histogram of *ammonium ion* is in Figure 1b. The intersection (Figure 1c) is computed as the minimum for each pair of argument values of the same character, with missing values in one argument omitted from the result.

For example, *ammonium* contains one “o” while there are two letters “o” in *ammonium ion.* As min(1, 2) = 1, the resulting histogram in Figure 1c contains the entry “1” for the letter “o”. As there is no blank in *ammonium,* there is also no entry for the blank character in the resulting histogram. In order to compute the size (the “absolute value” ||) of the histogram intersection in Figure 1c, the sum of all the numbers in the result matrix is calculated. For Figure 1c, the size of the histogram intersection is (1 + 1 + 3 + 1 + 1 + 1) = 8.

An example of three strings sharing the same prefix is shown in Table 2. Strings (1) and (2) comprise the first pair, and strings (1) and (3) form the second pair. Clearly, the first pair of strings is more similar than the second pair. To account for this and similar cases, the length of the approximately common prefix is divided by the average string length in formula (1). In Table 2, strings (1) and (2) belong to the UMLS concept with Concept Unique Identifier (CUI) C0002611, while string (3) is associated with (CUI) C1816069.

**Table 2:** UMLS terms sharing the same longest approximately common prefix

**#**	**String**	**Length**
1	Ammonium	8
2	Ammonium ion	12
3	AMMONIUM-CHLORIDE 1 MG/CYANOCOBALAMIN 5 MCG/FERRIC AMMON IUM CITRATE 40 MG/FOLIC ACID 1 MG/LYSINE HYDROCHLORIDE 100 MG/MAGNESIUM SULFATE 1 MG/MANGANESE SULFATE ANHYDROUS 1 MG/NIACIN 5 MG/PANTHENOL 1 MG/POTASSIUM SULFATE 1 MG/PYRIDOXINE HYDROCHLORIDE 0.5 MG/RIBOFLAVIN 1.2 MG/THIAMINE HYDROCHLORIDE 12 MG/ZINC SULFATE 1 MG ORAL LIQUID [HEMERGON]	369

The LACP algorithm is in Table 3. The algorithm begins by setting the histogram intersection at 0. The search for the longest approximately common prefix begins with the first character of each string. In steps 3 and 4, the characters at the current position *i* of strings *S* and *T* are added to the corresponding histograms. In steps 5 through 9, all characters in the histogram of string *S* are compared against the histogram of string *T* at the current iteration *i*. At this point, the search has advanced to the i-th character of each string. Steps 6 and 7 describe the following: when a character *c* is found in both histograms, operation Get(*c*) retrieves the count of this character from both *HistS* and *HistT*. Then the smaller of the two values is added to the intersection. The search continues until the parameter α is reached, as shown in line 9, or the last character of the shorter string is processed, as specified in line 2. In the latter case, the length of the shorter string is computed in line 11.

**Table 3:** Algorithm of the LACP method

**No**	**Line**	**Complexity**
1	*Intersection* = 0	O(1)
2	FOR *i* = 1 to min(|*S*|,|*T*|)	O(n)
	BEGIN	
3	*HistS*.add(*S*_*i*_)	O(1)
4	*HistT*.add(*T*_*i*_)	O(1)
5	FOR (Char *c* : *HistT*.Keyset())	Constant
	BEGIN	
6	IF *HistS*.ContainsKey(*c*)	O(1)
7	THEN *Intersection* =	O(1)
	*Intersection* + min(*HistS*.Get(*c*), *HistT*.Get(*c*))	
8	END	
9	IF (*i* - *Intersection*) = *α*	O(1)
	THEN RETURN $1-\frac{i-1}{\left(S.\mathrm{length}\left(\right)+T.\mathrm{length}\left(\right)\right)/2}$	
10	END	
11	RETURN $1-\frac{\min\left(\left|S|,|T\right|\right)}{\left(S.\mathrm{length}\left(\right)+T.\mathrm{length}\left(\right)\right)/2}$	O(1)
Total complexity		O(n)

Despite its linear time computational complexity, the simplicity of the LACP algorithm ensures a short execution time. The big-O computational complexity is commonly used for estimating the speed of an algorithm in computer science. The calculation of the LACP method time complexity is shown in Table 3. The inner loop in step 5 is bound by the number of printable characters and therefore constant [28]. Thus, the complexity of the LACP algorithm is linear, i.e., *O*(*n*), which is fast comparing to other algorithms evaluated in this paper.

### LACP-based interactive spell checker

We have employed the LACP method to develop an interactive online spell checker [29] for SNOMED CT terms. The spell checker is a program written in PHP, which connects to a MySQL database containing SNOMED CT terms from the 2009AB edition of the UMLS. The goal of the application is to evaluate LACP performance by revealing the set of SNOMED CT terms that are similar to the user-provided input term.

The spell checker accepts an input query and interactively outputs the SNOMED CT terms satisfying the condition *LACP*(*S*, *T*) < *t*. Here, *S* is the input term, *T* is a SNOMED CT term, and *t* is a threshold. To reduce the run time, the algorithm limits the set of search terms by applying length criteria as described below.

There are several parameters that define the performance of the spell checker depending on the mode of operation. The length of a SNOMED CT term |*T*| that is considered a potential match is bound by formulas (8), (10), and (11) in conformity with each of the three modes of operation. Parameters *A* and *B* are used in (11) to determine the values of the lower and upper limits for |*T*|, respectively. Parameter α sets the upper bound for the number of allowed character mismatches in the prefixes of strings *S* and *T*. Threshold *t* defines the “cutoff point” for the LACP score; a pair of strings *S* and *T* is considered to be a match when the LACP score is less than the threshold *t*.

Three modes of operation are implemented: (a) a search with dynamically estimated parameters; (b) a search with static parameters; and (c) a search with user-defined parameters. In case (a), the search is limited to the database terms meeting the criterion (5), while α is defined in (6) and threshold *t* is 0.1.

(5) $$\text{max}\;\left(0,\left|S\right|-\left\lceil \frac{\left|S\right|}{10}\right\rceil -3\right)<\left|T\right|<\left|S\right|+\left\lceil \frac{\left|S\right|}{10}\right\rceil +3$$

For example, for string *S* = Ischemia, |*S*| = 8. Thus, according to (5), the dynamic search would be limited to terms longer than 4 characters and shorter than 12 characters. In case (a), parameter α is set individually for each pair of strings *S* and *T* as shown in (6):

(6) $$\alpha =\left\lceil \frac{\text{min}\left(\left|S\right|,\left|T\right|\right)}{5}\right\rceil$$

In case (b), α is set to 1, threshold *t* is 0.1, and the length of a term should be in the following range (7):

(7) $$\text{max}\;\left(0,\left|S\right|-3\right)<\left|T\right|<\left|S\right|+3$$

In case (c), a user selects parameter values from predefined sets. The search is restricted to terms with lengths within the interval (8).

(8) $$\text{max}\;\left(0,\left|S\right|-A\right)<\left|T\right|<\left|S\right|+B$$

Parameters *A*, *B*, and α are constrained to integers in the interval 1..15, and threshold *t* must be selected from the set {0.1, 0.2, 0.3, 0.4, 0.5, 0.6, 0.7, 0.8, 0.9, 1.0}.

The dynamic search option adjusts the number of allowed misspellings α along with minimum and maximum term length parameters according to the input query. The dynamic search offers flexibility without user intervention. The threshold *t* is set to 0.1 for this search mode.

The static search option operates with constant parameter values. It allows only one misspelling. The lengths of the returned strings must be in the neighbourhood of ±3 characters of the input query length. This option decreases the search time for longer input terms compared to the dynamic search option.

The search mode based on user-defined parameters expands parameter options within pre-defined ranges. This mode is intended for users who are not satisfied with the results of the dynamic and static modes or who seek more refined results.

In summary, the dynamic option is suggested when results significantly vary in length from the search query. The static search option should be used when the resulting strings is expected to lie in the neighbourhood of the input term. The search with user-defined parameters is intended for fine-tuning results or for a more advanced search.

## Results

The LACP was compared to nine other well-known approximate string distance metrics: Jaccard [30], Jaro [31], Jaro-Winkler [32], Levenshtein [33], Monge-Elkan [34], Needleman-Wunsch [35], Smith-Waterman [36], TFIDF [37], and Soft TFIDF [15]. LACP was compared with these string matching methods on four datasets derived from Version 2009AB of the UMLS (Table 1). Dataset *D*_1_ was obtained by counting occurrences of each Concept Unique Identifier (CUI) within the UMLS [38], retrieving all terms corresponding to the 100 most frequent CUIs and eliminating records with duplicate terms. *D*_2_ was created in the same way, but limited to concepts from SNOMED CT [39]. *D*_3_ was built by retrieving the 5,000 longest terms from the multiple UMLS sources. *D*_4_ was constructed by taking the 5,000 longest terms from SNOMED CT.

SecondString [17], an open-source Java toolkit, was used as an experimental test bed. During the experiments, each term was matched against those within a set of candidate pairs. This type of set reduces the problem size and speeds up experiment execution. The candidate set includes pairs of terms from the dataset that share one or more common words. The goal was to determine whether every pair of terms has the same CUI. Using common performance evaluation methods from information retrieval [27], we calculated average precision (*P*), recall (*R*) and Maximum *F*_1_ values (formulae (9), (10), and (11)), and graphed precision-recall (P-R) curves for our method and for the competing techniques. Precision and recall are tradeoffs against one another: on the one hand, it is possible to obtain the maximum value of recall with a low value of precision by retrieving all documents for all queries. On the other hand, the precision usually decreases as the number of retrieved documents grows. A single measure that trades off precision versus recall is the *F* measure, which is the weighted harmonic mean of precision and recall [40].

(9) $$P=\frac{D_{r}}{D_{t}}$$

(10) $$R=\frac{D_{r}}{N_{r}}$$

(11) $$F_{1}=\frac{2P*R}{P+R}$$

In (9) and (10), *D*_*r*_ denotes the number of relevant items retrieved, *D*_*t*_ is the total number of retrieved items, and *N*_*r*_ is the number of relevant items in the collection.

LACP achieves the highest average precision for datasets *D*_1_ and *D*_4_ (Table 4) and the best values of Maximum *F*_1_ for *D*_1_, *D*_2_, and *D*_4_ (Table 5). TFIDF and Soft TFIDF achieve the best scores of average precision for *D*_1_ and *D*_2_ and the largest Maximum *F*_1_ for *D*_3_. It is worth noting that TFIDF and Soft TFIDF demonstrate exactly the same values of average precision and Maximum *F*_1_ for each dataset, although Soft TDIDF executes the operation at a significantly slower pace.

**Table 4:** Average precision P

**Dataset**	***D***_**1**_	***D***_**2**_	***D***_**3**_	***D***_**4**_
Jaccard	0.31	0.33	0.22	0.54
Jaro	0.26	0.40	0.14	0.69
Jaro-Winkler	0.44	0.45	0.14	0.69
Levenshtein	0.16	0.21	0.18	0.54
Monge-Elkan	0.22	0.32	0.12	0.65
Needleman-Wunsch	0.16	0.21	0.18	0.54
Smith-Waterman	0.18	0.16	0.09	0.34
TFIDF	0.51	***0.55***	***0.25***	0.69
Soft TFIDF	0.51	***0.55***	***0.25***	0.69
LACP	***0.62***	0.51	0.12	***0.84***

Note: The best values for each column are formatted in bold italics.

**Table 5:** **Maximum** ***F***_**1**_

**Dataset**	***D***_**1**_	***D***_**2**_	***D***_**3**_	***D***_**4**_
Jaccard	0.33	0.38	0.37	0.59
Jaro	0.33	0.49	0.28	0.77
Jaro-Winkler	0.56	0.57	0.28	0.77
Levenshtein	0.21	0.28	0.33	0.65
Monge-Elkan	0.24	0.37	0.26	0.67
Needleman-Wunsch	0.21	0.28	0.33	0.65
Smith-Waterman	0.21	0.22	0.18	0.38
TFIDF	0.49	0.58	***0.40***	0.70
Soft TFIDF	0.49	0.58	***0.40***	0.71
LACP	***0.69***	***0.61***	0.27	***0.92***

Note: The best values for each column are formatted in bold italics.

**Figure 2:**
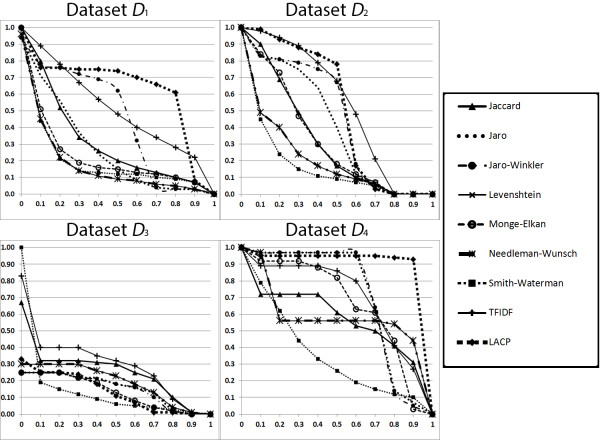
**Precision-recall curves of the evaluated methods.** Figure 2 depicts four precision-recall charts plotting interpolated precision values at 11 recall levels. The horizontal axis shows 11 recall points; the vertical axis displays interpolated precision values. A method with a larger area under its curve demonstrates a better result. The differences in performance between LACP, TFIDF and Soft TFIDF are easily apparent. For *D*_1_ and *D*_4,_ LACP consistently outperforms the other two methods. It is important to note, however, that on *D*_2_, LACP experiences a rapid precision drop after recall = 0.5, and that on *D*_3_, LACP is inferior to most methods.

Table 6 shows that LACP is the fastest method on every dataset. Figure 2 depicts four precision-recall charts plotting interpolated precision values at 11 recall levels [27]. The horizontal axis shows 11 recall points; the vertical axis displays interpolated precision values. A method with a larger area under its curve demonstrates a better result. The differences in performance between LACP, TFIDF and Soft TFIDF are easily apparent. For *D*_1_ and *D*_4,_ LACP consistently outperforms the other two methods. It is important to note, however, that on *D*_2_, LACP experiences a rapid precision drop after recall = 0.5, and that on *D*_3_, LACP is inferior to most methods.

**Table 6:** Execution time in seconds

**Dataset**	***D***_**1**_	***D***_**2**_	***D***_**3**_	***D***_**4**_
Jaccard	70	20	568	324
Jaro	105	25	3,637	1,102
Jaro-Winkler	115	26	3,617	1,265
Levenshtein	1,273	301	57,811	16,596
Monge-Elkan	6,240	1,340	258,502	77,555
Needleman-Wunsch	1,294	258	57,982	15,918
Smith-Waterman	1,444	293	58,753	17,519
TFIDF	132	37	928	558
Soft TFIDF	208	144	186,937	11,983
LACP	***40***	***11***	***202***	***233***

Note: The best values for each column are formatted in bold italics.

## Discussion

The primary advantage of the LACP method is its short execution times, a feature that is highly desirable when dealing with the large data sets involved in Medical Informatics. The performance of the LACP method can be interpreted by studying the structure of the datasets *D*_1_, -*D*_4_. Datasets *D*_1_, *D*_2_, and *D*_4_ have higher numbers of terms per concept compared to dataset *D*_3_ (see Table 1). Thus, *D*_1_, *D*_2_, and *D*_4_ have a higher number of records that have the same CUIs and have approximately common prefixes. This allows the LACP algorithm to outperform other more complicated well-known methods on *D*_1_, *D*_2_, and *D*_4_.

However, the LACP method performed poorly on *D*_3_. This is due to the large number of concepts with similar terms. As shown in Table 7, five terms share a 146-character-long common prefix, for example. By design, such terms are evaluated by LACP as very similar, which in fact is incorrect. Large numbers of such similarly spelled UMLS terms with different identifiers leave no chance for the LACP algorithm to succeed in these contexts.

**Table 7:** **Example of similar terms with different concept IDs from dataset** ***D***_**3**_

**CUI**	**Term**
C0602912	Yohimban-16-carboxylic acid, 11,17-dimethoxy-18-((3,4,5-trimethoxybenzoyl)oxy)-, methyl ester, (3beta,16beta,17alpha,18beta,20alpha)-, mixt. with 4-chloro-N(1)-methyl-N(1)-((tetrahydro-2-methyl-2-furanyl)methyl)-1,3-benzenedisulfonamide and 3-hydroxy-alpha-methyl-L-tyrosine
C0053099	Yohimban-16-carboxylic acid, 11,17-dimethoxy-18-((3,4,5-trimethoxybenzoyl)oxy)-, methyl ester, (3beta,16beta,17alpha,18beta,20alpha)-, mixt. with 4-chloro-N(1)-methyl-N(1)-((tetrahydro-2-methyl-2-furanyl)methyl)-1,3-benzenedisulfonamide and myo-inositol hexa-3-pyridinecarboxylate
C0050737	Yohimban-16-carboxylic acid, 11,17-dimethoxy-18-((3,4,5-trimethoxybenzoyl)oxy)-, methyl ester, (3beta,16beta,17alpha,18beta,20alpha)-, mixt. with 6-chloro-3,4-dihydro-2H-1,2,4-benzothiadiazine-7-sulfonamide 1,1-dioxide and 1(2H)-phthalazinone hydrazine
C0600796	Yohimban-16-carboxylic acid, 11,17-dimethoxy-18-((3,4,5-trimethoxybenzoyl)oxy)-, methyl ester, (3beta,16beta,17alpha,18beta,20alpha)-, mixt. with 6-chloro-3,4-dihydro-2H-1,2,4-benzothiadiazine-7-sulfonamide 1,1-dioxide and 5-ethyl-5-(1-methylpropyl)-2,4,6(1H,3H,5H)-pyrimidinetrione monosodium salt
C0602088	Yohimban-16-carboxylic acid, 11,17-dimethoxy-18-((3,4,5-trimethoxybenzoyl)oxy)-, methyl ester, (3beta,16beta,17alpha,18beta,20alpha)-, mixt. with 6-chloro-3,4-dihydro-2H-1,2,4-benzothiadiazine-7-sulfonamide 1,1-dioxide, 1(2H)-phthalazinone hydrazone and potassium chloride (KCl)

We note that the current online spell checker is a prototype. It has not been optimized for speed nor is it intended to compete with the well-known Google Instant Search [10], which displays search predictions as the user types a query. Instead, our goal is to create a spell checker specifically for use with biomedical terminologies. The remarkable difference between the excellent performance of LACP on datasets *D*_1_, *D*_2_, and *D*_4_ and its disappointing performance on *D*_3_ indicates that approximate string matching methods exhibit a certain degree of domain dependence. In fact, as detailed in an extensive research report by Rudniy [41], domain dependence has been shown to be a common phenomenon.

## Conclusions

LACP is a novel method we have developed for computing approximate string similarities based on assessing the length of approximately common string prefixes. The algorithm implements a normalization technique by dividing the length of the approximately common prefix by the average length of the pair of strings. LACP performed better than a number of well-known string similarity algorithms on three out of four datasets and demonstrated the shortest execution times on all four. For the average precision measure, LACP achieved the highest values of 0.62 on dataset *D*_1_ and 0.84 on dataset *D*_4_. On *D*_3_, LACP was second best, with an average precision of 0.51. Our method had the best values of Maximum *F*_1_ on three datasets: 0.69 on *D*_1_, 0.61 on *D*_2_, and 0.92 on *D*_4_. However, LACP experienced a drop in performance on dataset *D*_3_. In terms of execution time, LACP was on average two times faster than the Jaccard method, which achieved the second best times.

The LACP method demonstrated superior performance on certain types of biomedical datasets though its productivity has to be determined for other corpora. Another common limitation of the approximate string matching methods lies in the inability to determine that differently spelled synonyms correspond to the same concept. For such cases, either semantic methods or expert insight are required.

In future work, we will attempt to identify the cause and solve the problem of performance variability due to differences in dataset characteristics. Another branch of future research consists of investigating the best value for parameter α. The ultimate—though difficult—goal is to develop an approximate string matching method that recognizes and adapts to the distinctive characteristics of each dataset.

## Abbreviations

SNOMED CT: SNOMED clinical terms; UMLS: Unified medical language system; ASM: Approximate string matching; LACP: Longest approximately common prefix; TFIDF: Term frequency/inverse document frequency; CUI: Concept unique identifier.

## Competing interests

The authors declare that they have no competing interests.

## Authors’ contributions

AR, MS, and JG participated in the algorithm design and evaluation, and drafted the manuscript. All authors read and approved the final manuscript.

## References

[B1] Navarro G A guided tour to approximate string matching ACM Comp Surv 2001 33 1 31 88 10.1145/375360.375365

[B2] Yap TK Parallel computation in biological sequence analysis IEEE Trans Parallel Distrib Syst 1998 9 3 283 294 10.1109/71.674320

[B3] Sauleau EA Paumier J-P Buemi A Medical record linkage in health information systems by approximate string matching and clustering BMC Med Inf and Decision Making 2005 0 5 32 10.1186/1472-6947-5-32 PMC1274322 16219102

[B4] Huang KC Geller J Halper M Cimino JJ Teich JM, Suermondt J, Hripcsak G Piecewise synonyms for enhanced UMLS source terminology integration Proc. AMIA Annual Symp 2007 339 343 PMC2655905 18693854

[B5] Wang JF Assessment of approximate string matching in a biomedical text retrieval problem Comput Biology Medicine 2005 35 8 717 724 10.1016/j.compbiomed.2004.06.002 16124992

[B6] 2013AB UMLS Release Notes and Bugs http://www.nlm.nih.gov/research/umls/knowledge_sources/metathesaurus/release/notes.html [accessed 4.15.14]

[B7] SPECIALIST NLP Tools http://lexsrv3.nlm.nih.gov/Specialist/Home/index.html

[B8] MetaMap 2013 Usage http://metamap.nlm.nih.gov/Docs/Metamap13_Usage.shtml

[B9] Jonquet C Shah NH Musen MA The open biomedical annotator Summit Transl Bioinformatics 2009 2009 56 PMC3041576 21347171

[B10] Apache UIMA ConceptMapper annotator documentation http://uima.apache.org/downloads/sandbox/ConceptMapperAnnotatorUserGuide/ConceptMapperAnnotatorUserGuide.html

[B11] Funk C Baumgartner W Jr Garcia B Roeder C Bada M Cohen KB Hunter LE Verspoor K Large-scale biomedical concept recognition: an evaluation of current automatic annotators and their parameters BMC Bioinformatics 2014 15 59 10.1186/1471-2105-15-59 24571547 PMC4015610

[B12] Tan H Lambrix PA Method for recommending ontology alignment strategies The Semantic Web, Volume 4825 2007 Berlin: Springer 494 507

[B13] Tolentino HD Matters MD Walop W Law B Tong W Liu F Fontelo P Kohl K Payne DC A UMLS-based spell checker for natural language processing in vaccine safety BMC Med Inf and Decision Making 2007 7 3 10.1186/1472-6947-7-3 PMC1805499 17295907

[B14] Sahay S Agichtein E Li B Garcia E Ram A Kargupta H, Han J, Yu PS, Motwani R, Kumar V Semantic annotation and inference for medical knowledge discovery Proceedings of the NSF Symp. on Next Generation of Data Mining: 10-12 October 2007 2007 Baltimore: Chapman & Hall/CRC 101 105

[B15] Cohen W Ravikumar P Fienberg S Subbarao K, Craig A A comparison of string distance metrics for name-matching tasks Proceedings of Information Integration on the Web: 9-10 August 2003 2003 Acapulco, Mexico: Knoblock 73 78

[B16] Plaza L Diaz A Gervas P A semantic graph-based approach to biomedical summarization Artif Intell Med 2011 53 1 14 10.1016/j.artmed.2011.06.005 21752612

[B17] Zheng H-T Borchert C Jian Y A knowledge-driven approach to biomedical document conceptualization Artif Intell Med 2010 49 2 67 78 10.1016/j.artmed.2010.02.005 20371168

[B18] Song M Rudniy A Xue-wen C, Xiaohua H, Sun K Detecting duplicate biological entities using Markov Random Field-based edit distance Proceedings of 2008 IEEE International Conference on Bioinformatics and Biomedicine: 5-7 November 2008 2008 Philadelphia: IEEE Computer Society 457 460

[B19] Wellner B Castano J Pustejovsky J Jagadish HV Adaptive string similarity metrics for biomedical reference resolution Proceedings of the 13th International Conference on Intelligent Systems for Molecular Biology: 25-29 June 2005 2005 Detroit: David States and Burkhard Rost 9 16

[B20] Bodenreider O Burgun A Aligning knowledge sources in the UMLS: methods, quantitative results, and applications Stud Health Technol Inform 2004 107 327 331 15360828 PMC4303371

[B21] Yamaguchi A Yamamoto Y Kim JD Takagi T Yonezawa A Discriminative application of string similarity methods to chemical and non-chemical names for biomedical abbreviation clustering BMC Genomics 2012 13 Suppl 3 S8 22759617 10.1186/1471-2164-13-S3-S8 PMC3394426

[B22] Sauleau EA Paumier JP Buemi A Medical record linkage in health information systems by approximate string matching and clustering BMC Med Inform Decis Mak 2005 5 32 10.1186/1472-6947-5-32 16219102 PMC1274322

[B23] Zunner C Burkle T Prokosch HU Ganslandt T Mapping local laboratory interface terms to LOINC at a German university hospital using RELMA V. 5: a semi-automated approach J Am Med Inform Assoc 2013 20 293 297 10.1136/amiajnl-2012-001063 22802268 PMC3638185

[B24] McDonald C Huff S Deckard J Holck K Vreeman DJ Logical observation identifiers names and codes (LOINC) users’ guide [https://loinc.org/downloads/files/LOINCManual.pdf]

[B25] RELMA. regenstrief LOINC mapping assistant. version 6.2. Users’ manual Regenstrief Institute, Inc. and LOINC Committee 2013 http://loinc.org/downloads/files/RELMAManual.pdf

[B26] Parcero E Maldonado JA Marco L Robles M Berez V Mas T Rodriguez M Pedro Pereira R, Mykola P, João G, Ricardo Cruz C, Jiming L, Agma T, Peter L, Paolo S Automatic mapping tool of local laboratory terminologies to LOINC Proceedings of 26th IEEE International Symposium on Computer-Based Medical Systems: 20-22 June 2013 2013 Porto, Portugal: IEEE 409 412

[B27] Rudniy A Geller J Song M Shortest path edit distance for enhancing UMLS integration and audit Proceedings American Medical Informatics Association Annual Symposium: 13-17 November 2010 2010 Washington, D.C.: AMIA 697 701 PMC3041450 21347068

[B28] Maini AK Digital electronics: principles, devices and applications 2007 Hoboken, NJ: Wiley

[B29] SNOMED CT Spell Checker 2013 http://snomedct-spell-checker.com> [accessed 10.30.2013]

[B30] Jaccard P The distribution of the flora in the alpine zone New Phytol 1912 11 2 37 50 10.1111/j.1469-8137.1912.tb05611.x

[B31] Jaro MA Advances in record-linkage methodology as applied to matching the 1985 Census of Tampa Florida J Amer Stat Assoc 1989 89 414 420

[B32] Winkler WE String comparator metrics and enhanced decision rules in the Fellegi-Sunter model of record linkage Proc Section Survey Research Methods Amer Stat Assn 1990 354 359

[B33] Levenshtein VI Binary codes capable of correcting deletions, insertions and reversals Sov Phys Dokl 1966 10 707 710

[B34] Monge AE Elkan CP The field matching problem: algorithms and applications 1996 Discovery and Data Mining: Proc. Second Int. Conf. on Knowl

[B35] Needleman SB Wunsch CD A general method applicable to the search for similarities in the amino acid sequence of two proteins J Mol Biol 1970 48 443 453 10.1016/0022-2836(70)90057-4 5420325

[B36] Smith TD Waterman MS Identification of common molecular subsequences J Mol Biol 1981 147 195 197 10.1016/0022-2836(81)90087-5 7265238

[B37] Salton G Buckley C Term weighting approaches in automatic text retrieval Inf Process Manage 1988 24 5 513 523 10.1016/0306-4573(88)90021-0

[B38] Humphreys BL Lindberg DAB Schoolman HM Barnett GO The unified medical language system: an informatics research collaboration J Am Med Inform Assoc 1998 5 1 1 11 10.1136/jamia.1998.0050001 9452981 PMC61271

[B39] IHTSDO: SNOMED CT 2013 http://www.ihtsdo.org/snomed-ct> [accessed 10.30.2013]

[B40] Manning C Raghavan P Schütze H Introduction to information retrieval 2008 Cambridge, England: Cambridge University Press

[B41] Rudniy A Approximate string matching methods for duplicate detection and clustering tasks Ph.D. Dissertation 2012 Newark, NJ: CS dept., NJIT

